# Mechanisms underlying lactic acid tolerance and its influence on lactic acid production in *Saccharomyces cerevisiae*

**DOI:** 10.15698/mic2021.06.751

**Published:** 2021-04-14

**Authors:** Arne Peetermans, María R. Foulquié-Moreno, Johan M. Thevelein

**Affiliations:** 1 Laboratory of Molecular Cell Biology, Institute of Botany and Microbiology, KU Leuven, Flanders, Belgium.; 2 Center for Microbiology, VIB, Kasteelpark Arenberg 31, B-3001, Leuven-Heverlee, Flanders, Belgium.; 3 NovelYeast bv, Open Bio-Incubator, Erasmus High School, Laarbeeklaan 121, 1090 Brussels (Jette), Belgium.

**Keywords:** lactic acid tolerance, Saccharomyces cerevisiae, lactic acid production, yeast cell factory, lactic acid stress response

## Abstract

One of the major bottlenecks in lactic acid production using microbial fermentation is the detrimental influence lactic acid accumulation poses on the lactic acid producing cells. The accumulation of lactic acid results in many negative effects on the cell such as intracellular acidification, anion accumulation, membrane perturbation, disturbed amino acid trafficking, increased turgor pressure, ATP depletion, ROS accumulation, metabolic dysregulation and metal chelation. In this review, the manner in which *Saccharomyces cerevisiae* deals with these issues will be discussed extensively not only for lactic acid as a singular stress factor but also in combination with other stresses. In addition, different methods to improve lactic acid tolerance in *S. cerevisiae* using targeted and non-targeted engineering methods will be discussed.

## BACKGROUND

The compound lactic acid is a molecule with a long history of use in the food industry as a preservative, pH buffering agent, flavoring agent and acidulant [1]. More recently it has also been used in other industries such as the pharmaceutical, cosmetic, textile and most importantly the chemical industry [2]. In the chemical industry, lactic acid is used as a platform molecule to produce a large variety of chemicals including acrylic acid, propylene glycol, acetaldehyde, 2,3-pentanedione, pyruvic acid, and the lactide molecule which is used to make the polymer poly-lactic acid (PLA) [3]. Currently, the rising demand for the bio-based, biodegradable plastic PLA is the main contributor to the rising demand for lactic acid [4, 5].

Presently, lactic acid is mainly produced with microbial fermentation, using lactic acid bacteria. However, because of their relatively high acid sensitivity, neutralizing agents such as CaCO_3_ have to be added during fermentation for optimal production. Because of this, lactic acid is produced in its salt form (calcium lactate) due to the pH of the fermentation broth being higher than the pKa of lactic acid. Lactic acid is freed from its salt by recovery with sulfuric acid, which causes the calcium ion from calcium lactate and the sulfate group from sulfuric acid to react leading to the formation of calcium sulfate or gypsum which is an undesirable by-product made in very large quantities [3].

With the aim of achieving the production of lactic acid in its undissociated acid form, researchers have been metabolically engineering lactic acid bacteria for increased tolerance to low pH, achieving some promising results, inherently, weak-acid tolerant yeast species have been metabolically engineered to produce lactic acid. Yeast species are not only good hosts for lactic acid production because of their higher tolerance to low pH, but also because of their simple nutrient requirements in contrast to lactic acid bacteria, which facilitates down-stream processing [7–9]. Yeast species that have already been engineered for lactic acid production include *Saccharomyces cerevisiae* [10–14]*, Schizosaccharomyces pombe* [15]*, Zygosaccharomyces bailli* [16]*, Candida sonorensis* [17]*, Candida krusei* (also named *Candida glycerinogenes, Pichia kudriavzevii* and *Issatchenkia orientalis*) [18, 19]*, Candida boidinii* [20]*, Pichia stipitis* [21]*, Pichia pastoris* [22]*, Kluyveromyces marxianus* [23, 24] and *Kluyveromyces lactis* [24, 25]. Of all these species, *S. cerevisiae* has been most extensively studied as it is relatively easy to genetically engineer, because of the availability of the complete genome sequence of many strains and of a wealth of molecular information on gene products and metabolic pathways. Additionally, its fermentation and process technology for large-scale production is well established, making it attractive to use under industrial conditions.

However, despite the higher inherent tolerance of these yeast species, growth inhibition due to lactic acid accumulation is still the most limiting factor preventing high-titer production of lactic acid [8, 10, 26, 27]. To circumvent this, the yeast species should be made even more lactic acid tolerant by metabolic engineering strategies. Targeting of the mechanisms responsible for coping with lactic acid stress has shown to be effective in increasing the titers, productivity and yield of lactic acid producing *S. cerevisiae* strains. Genes involved in lactic acid tolerance that have been targeted to improve lactic acid titers in *S. cerevisiae* strains include *GSF2* [10], *HAA1* [28]*, SSB1* [13]*, SAM2 [26], ESBP6* [29]. Moreover, Suzuki *et al.* showed with the quadruple deletion strain *dse2*Δ *scw11*Δ *eaf3*Δ *sed1*Δ that combinatorial disruption of multiple genes which individually improve lactic acid tolerance can result in a further improvement of performance [27]. Moreover, deletion of these four genes together increased lactic acid productivity by 27%, while individual mutations did not result in increased productivity [27].

We can conclude that lactic acid tolerance is a crucial factor, which must be considered when high titers of lactic acid are to be obtained. Unravelling the mechanisms underlying the lactic acid stress response can help us identify genetic targets that can be modified to improve the productivity of lactic acid producing yeast species. In this review the mechanisms underlying lactic acid tolerance in *S. cerevisiae* and the use of this knowledge to develop high-performance industrial lactic acid producing *S. cerevisiae* strains will be discussed.

## THE DETRIMENTAL EFFECTS OF WEAK ORGANIC ACID STRESS

It is known that the presence of lactic acid or other weak organic acids causes severe growth defects in microorganisms. The degree of microbial inhibition by weak acids is largely determined by their chemical properties, in particular their pKa, their lipophilicity, and their volatility. Other factors besides the chemical properties of the weak acids playing a role are the pH of the medium and the concentration of the acid [30–33].

At external pH below the pKa of the weak acid, the acid will be predominantly in its undissociated, uncharged, and thus more lipophilic form. Because of its lipophilicity the undissociated acid can transverse the plasma membrane. Once inside the cell, the weak acid dissociates because of the neutral cytosolic pH. This dissociation causes a cascade of reactions inside the cell [33–36]. Because of its charged nature the anion cannot diffuse back out of the cell resulting in accumulation of the anion in the cytosol. The accumulated anions can intercalate with cellular membranes causing their permeabilization while also perturbating the function of the membrane imbedded proteins. Permeabilization of the plasma membrane causes the influx of ions and other small metabolites which leads to the stimulation of proton import, causing a dissipation of the electrochemical potential which is one of the driving forces of secondary transport. This results in the generation of an abnormally large turgor pressure and also inhibits normal membrane trafficking, which affects the uptake of many essential components including amino acids [31, 34, 37]. Another effect caused by the accumulation of weak acid anions in the cytosol, is the disruption of the electron transport chain on the mitochondrial inner membrane causing ATP depletion and the accumulation of reactive oxygen species (ROS) [37, 38]. Additionally, the increasing number of protons entering the cell due to plasma membrane perturbation together with the protons originating from the dissociated acid cause intracellular acidification. Intracellular acidification has an impact on a lot of cell functions including DNA and RNA synthesis and many metabolic processes. In *S. cerevisiae*, one of the metabolic processes that is disrupted by intracellular acidification is glycolysis [39–41]. Lower ATP generation due to disruption of glycolysis in addition with disruption of the energy-transport chain can partly explain the energy depletion in cells in response to weak acid addition [42]. Other possible causes include ATP-dependent export of protons and anions through the H^+^-ATPase and ABC pumps, respectively. Another effect associated with weak acid stress includes endoplasmic reticulum stress and the ensuing induction of the unfolded protein response [43]. Although the unfolded protein response is activated upon stress derived from most weak acids such as sorbic acid, propionic acid and acetic acid, it is not triggered by lactic acid stress [43]. Additionally, the chelation of metal ions by the negatively charged anions has the potential to limit metal availability in the growth medium [31].

It can be concluded that weak acid stress has a negative influence on many cellular functions. Because of the huge burden that weak acids exert on the cells, cells have developed diverse mechanisms to cope with this stress.

## THE TRANSCRIPTOMIC RESPONSE FOLLOWING LACTIC ACID STRESS IN *S. CEREVISIAE*

As discussed above, the effects exerted by weak organic acid stress on the cells is not only dependent on the type of microorganism but also depends on the chemical properties of the weak acid. Therefore, it is only logical that the coping mechanisms induced by the cell upon weak acid stress vary depending on the type of weak acid. In this chapter the transcriptomic response upon lactic acid stress will be discussed in detail.

Although the genes induced in response to weak acid stress do not coincide much for different types of acids, the same biological functions are affected: protein folding, lipid metabolism, cell-wall function and multidrug resistance [31]. The transcriptional response upon weak acid stress largely depends on the lipophilicity of the weak acid. Only one gene has been shown to be induced in response to all weak acids, which is the gene *TPO3*encoding for a H^+^-antiporter involved in multidrug resistance. This indicates that the existence of a general weak-acid response is limited [31].

The stress response upon addition of hydrophilic weak acids such as acetic acid and lactic acid is largely regulated by the Haa1-regulon [31, 36, 44–46]. It has been shown that Haa1-regulation of lactic acid stress is mainly exerted at low pH, meaning that Haa1 is primarily involved in the response to the undissociated form [47]. Upon lactic acid stress at low pH, Haa1 becomes localized to the nucleus where it is presumably activated due to phosphorylation and subsequently induces the expression of its target genes [46]. Activation of Haa1 is mediated by direct binding with the weak acid anion [36]. The return of Haa1 back to the cytosol is most likely due to Msn5-mediated export [46]. It was shown that *HAA1* overexpression in a lactic acid producing cell results in increased lactic acid productivity [28]. However, disruption of some of the most prominent Haa1 targets such as *TPO2, TPO3, YGP3* and *YRO2* did not cause a growth defect in high lactic acid concentrations [47]. This suggests a complex regulation network in which the deletion of one gene is compensated by differential expression of other elements in this network that deals with lactic acid stress.

In contrast, at pH 5, where lactate is the predominant form, the main transcriptional response in *S. cerevisiae* is related to iron homeostasis. This is mainly regulated by the transcription factor Aft1. Upon lactate stress at pH=5, Aft1 is translocated to the nucleus where it induces expression of its target genes [47, 48].

## ADAPTIVE RESPONSE TO INTRACELLULAR ACIDIFICATION DUE TO LACTIC ACID STRESS

Intracellular pH (pHi) homeostasis is a critical element in cell survivability. In *S. cerevisiae,* pHi homeostasis is tightly regulated by the proton translocating ATPase pump Pma1 present in the plasma membrane and vacuolar ATPase pumps (V-ATPases) in the vacuolar membrane [31, 49].

When intracellular acidification occurs due to weak acid stress, yeast cells rely primarily on stimulation of the H^+^-ATPase Pma1 to pump protons out of the cytosol. The upregulation of Pma1 is not attributed to increased synthesis of the enzyme but rather to post-transcriptional modification [50–52]. It has been shown that phosphorylation of Pma1, causes an increased affinity for ATP and an increase in maximum initial velocity (V_max_). Hence upon phosphorylation, more protons can be pumped out of the cell membrane, restoring pH homeostasis [53].

The role of the V-ATPase pumps in maintaining pHi homeostasis upon weak acid stress is the sequestering of protons into the lumen of the vacuole. Additionally, it has also been shown that V-ATPase activity plays a role in the correct trafficking and sorting of Pma1 to the plasma membrane [49].

It has been shown that the degree of intracellular acidification is largely dependent on the nature of the weak acid. At growth inhibitory concentration, lipophilic acids such as sorbic and benzoic acid do not cause a severe reduction in pHi, indicating a minor role of intracellular acidification in growth inhibition by these acids. In contrast, the more hydrophilic acetic acid molecule is known to cause long-term intracellular acidification and thus strong activation of the Pma1 proton pump [54, 55]. In agreement with these findings, when *S. cerevisiae* cells were exposed to lactic acid stress during growth, pHi dropped considerably [56]. However, in contrast to the acetic acid response, a decrease instead of an increase was seen in Pma1 activity upon lactic acid stress. This decrease in activity was attributed to a reduction in unsaturated fatty acid levels due to lactate anion stress. A reduction in unsaturated fatty acids affects the membrane fluidity and thus also the positioning and stability of Pma1 [55, 57, 58].

## EXPORT OF LACTATE AS A FACTOR IN LACTIC ACID STRESS

To counteract lactate-anion accumulation inside the cells, anions have to be exported out of the cells. Two lactate-anion transporters have been described in *S. cerevisiae.* The most prominent one is Jen1, a member of the major facilitator superfamily belonging to the sialate:proton symporter family [8, 57, 59, 60]. The other one is Ady2, a member of the acetate uptake family [59, 61–63].

### The role of the anion-proton symporter Jen1 in lactate transport

The most prominent type of lactate transporters found in microorganisms are lactate-proton symporters in which one or more protons are transported per anion. In *S. cerevisiae*, the monocarboxylate transporter Jen1 mediates lactate-proton symport. The stoichiometry of Jen1 in *S. cerevisiae* is one proton per anion. This implies an electroneutral transport that does not affect pH homeostasis in lactic acid producing cells [57, 64]. Jen1 is a transporter involved not only in the transport of lactic acid but also in transport of other monocarboxylic acids such as pyruvate, acetate and propionate [57, 59, 65], the micronutrient selenite [66] and the anticancer drug 3-bromopyruvate [67].

The expression of *JEN1* depends largely on the growth medium. When cells are grown in glucose (and formic or propionic acid), the transcription of *JEN1* is repressed [65]. In contrast, when cells are grown in lactic acid (and pyruvic acid or acetic acid) without glucose supplemented in the medium, *JEN1* is highly expressed leading to the import and consumption of the acids [65]. In agreement with these findings, it has been shown that overexpression of *JEN1* results in increased (inward) transport of lactic acid, while the deletion of *JEN1* causes impaired growth on lactic acid [68]. Interestingly, high expression of *JEN1* was also seen in cells grown in xylose and cellobiose media. In xylose-grown cells, *JEN1* was 55 times more expressed than in glucose-grown cells [62, 69]. Similarly, in cellobiose-grown cells, *JEN1* was five times more expressed [70].

The repression of *JEN1* when glucose is present is mainly mediated by transcription factors Mig1 and Mig2 [71]. In the cytosol, Mig1 and 2 are activated by dephosphorylation through the Reg1/Glc7 phosphatase and subsequently transported into the nucleus where they cause repression of *JEN1* [72]. In lactate medium, the kinase Snf1 phosphorylates Mig1 resulting in the translocation of the Mig1/2 complex from the nucleus to the cytosol, causing derepression of *JEN1* and activation of the Cat8 and Adr1 transcription factors. The Hap2/3/4/5 complex together with Cat8 and Adr1 act together to upregulate the *JEN1* gene among other genes that are involved in the utilization of non-fermentable sources [71, 73–75]. However, *JEN1* expression is not only mediated at the transcriptional level but also at the post-transcriptional level. Upon addition of glucose to lactic acid-grown cells, *JEN1* mRNA decay is triggered [76]. The underlying mechanism mediating this mRNA decay seems to be largely depending on the Dhh1 RNA helicase and the Pat1-Lsm decapping enhancers [75, 77]. Additional regulation of Jen1 is mediated at the protein level. Upon addition of glucose to lactic-acid grown cells, the loss of Jen1 is triggered via endocytosis and subsequent vacuolar degradation [78]. This is mediated by activation of Rod1 and the deactivation of Snf1 via the Glc7/Reg1 complex. The Glc7/Reg1 complex dephosphorylates Rod1, resulting in its release from the interaction with the 14-3-3 proteins and subsequent ubiquitination by Rsp5. The ubiquitinylated Rod1/Rsp5 complex mediates ubiquitination of Jen1 resulting in its degradation [75, 79].

Initially, Jen1 was only described as an inward transporter of lactate. However, the finding that overexpression of *JEN1* increases lactic acid production and yield in a lactic acid producing *S. cerevisiae* strain, led to the suggestion that Jen1 also has an export function [59, 80, 81]. Because Ldh activity drops considerably upon lactate-anion accumulation in the cytosol, increased export due to overexpression of *JEN1* fits with the increased lactic acid yield and productivity. The accumulation of only 10 g/L in the cytosol reduces Ldh activity by about 40% [80]. However, one thing to note is that the overexpression of *JEN1* only helps to a certain extent. The effect of the overexpression becomes negligible in high concentrations of lactate, suggesting possible saturation of Jen1 [80]. Additionally, upon glucose depletion, the extracellular lactic acid levels drop considerably in the lactic acid producing *JEN1*-overexpression strain. This was not seen in the lactic acid producing *JEN1*-deletion strain [59]. Taking this together we can conclude that Jen1 exports lactate out of the cell upon accumulation of the anion in the cytosol when glucose is still present while it imports lactate upon depletion of glucose at the end of the lactic acid fermentation.

In contrast to lactic acid fermentation in glucose media, overexpression of *JEN1* in xylose media does not improve lactic acid production. This is probably because *JEN1* is already highly expressed in xylose media [62].

### The role of acetate permease Ady2 in lactate transport

Similar to Jen1, the acetate transporter Ady2 is a proton-anion symporter that is also involved in lactic acid transport [64, 65, 82, 83]. In addition to lactic acid, Ady2 also transports acetate, propionate, ammonia, formate and pyruvate [59, 61, 63, 84].

The *ADY2* gene is induced by non-fermentable carbon sources and xylose and repressed by glucose, similar to the *JEN1* gene [62]. In xylose and cellobiose media the expression of *ADY2* in *S. cerevisiae* cells was seven and two times higher, respectively, than in glucose-media [69].

In an *S. cerevisiae* strain producing lactic acid from glucose, deletion of *ADY2* resulted in lower extracellular lactic acid concentrations in comparison with the strain with constitutive expression of *ADY2.* However, upon glucose depletion more lactic acid was consumed in the strain expressing *ADY2* [59]. These results suggest that the transport of lactate by Ady2 is bidirectional and that *ADY2* can be modified to increase lactic acid production in *S. cerevisiae* in glucose medium. This possibility was further supported by de Kok *et al.* [63]. They performed adaptive evolution for growth in lactic acid medium in a *JEN1-*deletion strain to find alternative lactate transporters in *S. cerevisiae.* This adapted strain was whole-genome sequenced to find the causative elements for its improved growth in lactic acid media. The causative elements found were two single nucleotide polymorphisms (SNPs) in *ADY2*, and a chromosomal rearrangement resulting in two extra copies of *ADY2.* The SNPs presumably caused extra space in the translocation site [63]. Interestingly, when the mutated *ADY2*-alleles were transformed into a *JEN1/ADY2*-deletion strain, biomass formation was drastically improved (41%). The substantial increase in biomass formation in the strain in which transport is mediated by the Ady2 isoform in contrast to the reference strain in which lactic acid transport is mainly mediated by Jen1, suggests a different mode of energy coupling of lactate transport by these Ady2 isoforms [63].

Single deletion of *ADY2* (and *JEN1*) did not affect lactic acid production from xylose in a lactic acid producing *S. cerevisiae* strain. However, the deletion of both *ADY2* and *JEN1* decreased lactic acid yield by 36% [62]. This implies a compensatory transcriptional regulation effect of the lactic acid transporters. When *JEN1* is deleted, *ADY2* transcription is upregulated and vice versa. Additionally, de Kok *et al.* [63] found that in contrast to what was seen by Casal *et al.* [65], *ADY2* could rescue growth of *JEN1*-deletion mutants in lactic acid media when *ADY2* was highly expressed from a plasmid [63]. Taken together, this suggests that Ady2 can take over the function of Jen1 despite its lower affinity for lactate (7 times lower [59]) provided that the expression of *ADY2* is many times higher than the *JEN1* expression level.

### Alternative export mechanisms of lactate in *S. cerevisiae* upon lactic acid stress

It has been shown that the deletion of both *ADY2* and *JEN1* decreases extracellular lactic acid production in a lactic acid producing heterolactic *S. cerevisiae* strain both in xylose and glucose media. However, lactate export is still operational in these deletion-strains, suggesting the presence of other lactate transporters in *S. cerevisiae* [59, 62, 82]. In addition, it has been suggested that under industrially relevant conditions (low extracellular pH, high extracellular lactic acid concentration), export of lactate with lactate-proton symport is no longer thermodynamically feasible [57]. Since lactate-proton symport is the mechanism by which both Ady2 and Jen1 work, other transport mechanisms should be present in *S. cerevisiae* that mediate export of lactic acid/lactate under these harsh conditions. The only mechanisms of export that are feasible under these conditions are all energy-requiring [57]. In line with this, it was found that export of lactic acid in *S. cerevisiae* strains producing lactic acid and no ethanol at all (homolactic) is energy-dependent [85]. Van Maris *et al.* showed that fermentation rate and growth of homolactic *S. cerevisiae* strains under anaerobic conditions was almost negligible. This was attributed to a zero net ATP yield, causing energy depletion by maintenance metabolism [85, 86]. Energy depletion abrogates protein synthesis, central carbon metabolism and any other energy consuming process [70, 86]. Van Maris *et al.* (2004) suggested that the net zero ATP yield was due to the need of one ATP for every molecule of lactate that is exported while the formation of one lactate molecule yields only one ATP molecule during glycolysis under anaerobic conditions [85].

Three different mechanisms can be the underlying cause for the energy-dependent lactate transport. The first one being energy-requiring primary transport of lactate out of the cell. Alternatively, facilitated diffusion of the lactate anion accompanied by export of the associated proton with a H^+^-ATPase pump could be the causative mechanism. Another possibility could be the antiport of lactate with a cation (Na^+^ or H^+^) followed by the subsequent cation extrusion with P-type ATPase pumps to maintain proton-motive force [82].

A primary (active) exporter of lactate has not been found yet in *S. cerevisiae*. In contrast, for other carboxylic acids such as sorbic, propionic, and benzoic acid, the ABC-transporter Pdr12 has been shown to be responsible for transport. Pdr12 is a substrate-specific transporter that can transport carboxylic acids with an aliphatic chain ranging from one to seven. However, highly hydrophobic (aliphatic chain length higher than seven) or more hydrophilic (lactic, acetic and formic) acids cannot be transported by Pdr12 [34, 87–89]. Nevertheless, it has been shown that the overexpression of *PDR12* makes *S. cerevisiae* cells more sensitive to lactic acid stress showing that it does play some kind of role in coping with lactic acid stress [87]. Additionally, when Hirasawa *et al.* (2013) expressed an *LDH* gene in 4802 deletion strains, the *PDR12* deletion strain was the only deletion strain showing a relevant change in lactic acid titer (>90% reduction) [82, 90]. However, this change in lactic acid titer was attributed to the auxotrophic strain background [34, 82].

An interesting concept proposed by Burgstaller *et al.* was that the low pH in the culture broth could be the driving force for weak-acid anion export. At low external pH, the concentration of dissociated acid is high inside the cell and low outside the cell. Hence the dissociated acid leaves the cell, following its concentration gradient by facilitated passive diffusion [91]. The ATP depletion in this type of transport is attributed to the use of the H^+^-ATPase pump (stoichiometry of 1 H^+^/ATP in *S. cerevisiae* [92, 93]) to export the remaining H^+^ of the lactic acid and maintain the membrane potential [85, 86]. At the moment, export of lactate via facilitated diffusion has not been found yet in *S. cerevisiae.* In contrast, facilitated diffusion of acetate was shown to be mediated by the aquaglyceroporin Fps1. However, the uptake of acetate dropped drastically at high pH, suggesting that the substrate is the undissociated acetic acid rather than the anion [94].

As previously suggested, the energy-dependent lactate export mechanism responsible for ATP-depletion in homolactic *S. cerevisiae* cells could be lactate-cation antiport combined with proton expulsion. This could be mediated by the Haa1-regulated Tpo2 and Tpo3 antiporters. Upon lactic acid addition both *TPO2* and *TPO3* showed upregulation, suggesting a role in lactic acid tolerance [47]. However, double deletion did not lead to impaired growth in lactic acid media. This could imply that there is a redundancy of lactate exporters or that Tpo1 and Tpo2 are not involved in lactate transport. However, an interesting finding by Fernandes *et al.* was that upon deletion of *TPO2*, acetate accumulates in the cell, showing the importance of Tpo2 in export of more hydrophilic organic acids [44].

With the aim of finding mechanisms involved in lactate export, Mans *et al.* performed a functional analysis study. Twenty-five putative lactate exporters including the genes encoding Jen1, Ady2, Pdr12, Fps1, Tpo2/3 [82] were deleted. In this 25-deletion strain an *LDH* gene derived from *Lactobacillus casei* was expressed and the strain tested for anaerobic growth. They found that in anaerobic glucose-grown batch cultures the 25-deletion strain exhibited a lower specific growth rate and biomass-specific lactate production compared to the control. This finding was attributed to a lower glycolytic flux. In contrast, in anaerobic glucose-limited chemostat cultures the lactate production rate was the same for the 25-deletion and the control strain, suggesting that one or more alternative transporters must be able to sustain lactic acid export [82].

We can conclude that Jen1 and Ady2 are probably not the only mechanisms involved in transport of the lactate anion. Most likely one or more ATP-dependent mechanisms are involved in lactate transport. Possibly the mechanisms mediating lactate transport are not as straightforward as one might think. There is a possibility that the export is mediated differently for different concentrations of lactic acid stress. It could be that the export of lactate in a medium with low lactic acid concentration is mainly mediated by neutral transport by transporters such as Jen1/Ady2. On the other hand, upon high lactic acid stress, other energy-requiring export mechanisms could be upregulated because in contrast to these neutral transport mechanisms, they are not hindered by thermodynamic constraints under these harsh conditions [57]. Therefore, it might be worthwhile to characterize lactic acid stress responses upon addition of very high lactic acid concentrations (>50 g/L lactic acid). More specifically, the lactic acid response in homolactic *S. cerevisiae* strains producing very high lactic acid titers should be investigated. It has been shown that cells producing low levels of a weak acid have time to adapt to the weak-acid stress in contrast to cells which are exposed rapidly to high concentrations of weak acid resulting in a different type of stress response [30]. A similar situation might be applicable to yeast producing the very high lactic acid titers required in industrial practice.

## AMINO ACID METABOLISM UPON LACTIC ACID STRESS IN *S. CEREVISIAE*

It is known that lactic acid stress results in a substantial decrease of intracellular amino acids [95]. An explanation could be the disruption of the proton gradient due to membrane perturbation, which is known to be the driving force for the amino acid transporters [96]. An alternative explanation could be the disturbance of vacuolar integrity. This could lead to incorrect localization of amino-acid transporters and decreased recycling of membrane proteins [95, 97]. Moreover, vacuolar integrity disturbance could have an effect on amino acid storage in the vacuole [98–100]. Additionally, it also affects peptide transport and hydrolysis, which are used as a source for amino acids [95, 101, 102]. The starvation of amino acids causes a general reduction in protein synthesis thereby affecting many cellular processes including stress-response [95]. In addition, amino acid depletion is also known to disturb intracellular pH homeostasis due to a decrease in glutamate and arginine decarboxylation which is a proton consuming process. Furthermore, amino acid depletion is also responsible for increased accumulation of oxygen radicals, resulting in additional stress [103].

To counteract amino acid depletion upon lactic acid stress, the cell reacts with upregulation of genes responsible for amino acid homeostasis [95]. The upregulation of genes responsible for biosynthesis and uptake of amino acids upon stress triggered by acetic acid or 2,4-dichlorophenoxyacetic acid is mediated by the TOR-pathway, suggesting that for the lactic acid stress response the same mechanism might be involved [45, 104]. Other responses induced upon lactic acid stress include vacuolar acidification, which helps with the stable location of Pma1. But also vacuolar fragmentation, which is suggested to help increase the surface to volume area of the vacuole resulting in more space for H^+^-ATPases and anion pumps, results in? more efficient efflux of protons and anions from the cytosol [95]. These adaptations help the cell to repair the proton gradient, resulting in more efficient amino acid uptake.

In contrast to what is seen for most amino acids, lactic acid stress causes an increase in some specific amino acids such as proline, cysteine, asparagine and glutamine. The increase in cysteine and glutamine levels has been attributed to increased glutathione (GSH) synthesis under lactic acid stress. GSH is synthesized from glutamine, glycine and cysteine for the purpose of ROS scavenging to counteract oxidative stress [105]. The increase in proline levels upon lactic acid stress has also been shown to have a role in lactic acid tolerance [105]. Currently, it is not exactly known how proline helps in coping with lactic acid stress. However, it has been suggested that it could be due to protecting the cell from a pHi drop [105]. Alternatively, it was suggested that it is due to oxidative stress protective properties of proline because increased proline levels trigger overexpression of *MPR1,* which is known to lead to decreased ROS levels and increased maintenance of cell viability [106, 107], or it might be due to ROS scavenging properties of proline itself [108].

## IRON METABOLISM IS INVOLVED IN LACTIC ACID TOLERANCE IN *S. CEREVISIAE*

Not only amino acid homeostasis but also metal cation homeostasis is involved in lactic acid tolerance. The expression of genes involved in iron metabolism in particular is the most affected upon lactic acid stress. Iron metabolism is mainly regulated by the transcription factor Aft1. Induction of Aft1 upon lactic acid stress and subsequent alteration in the expression levels of iron permeases, iron reductases, siderophore transporters, ferroxidases and siderophore uptake-related proteins shows the crucial role iron metabolism plays in lactic acid tolerance [48]. A possible explanation for the alternative regulation of iron metabolism upon lactic acid stress could be that iron transport is inhibited or that more iron is needed in the cell [48]. The latter could be the result of possible chelating abilities of iron. The positively charged cations can chelate the negatively charged lactate anions, reducing anion accumulation [31]. A possible counterargument to this hypothesis is the fact that the iron-lactate complex is able to interact with H_2_O_2_ and thereby enhance hydroxyl-radical generation via the Fenton reaction, which generates oxidative stress [109, 110].

## ENERGY METABOLISM IN *S. CEREVISIAE* UPON LACTIC ACID STRESS

It is known that weak acid stress has a negative influence on energy metabolism. It leads to disruption of the electron transport chain [37, 38], disruption of metabolic pathways responsible for ATP generation, such as glycolysis [39–41], and of the energy-requiring export of protons and anions [111].

Indeed, when cells are subjected to moderate weak acid stress, ATP levels decrease. However, upon high levels of weak acid stress, it was found that ATP levels increased [42]. The addition of 14 g/L of lactic acid (pH≈2.5) to the culture resulted in a 1.34 (±0.24) ATP/ADP ratio, which is a substantial increase in comparison with the control which was 0.23 (±0.05) [105]. The increase in ATP was mainly attributed to a decrease in H^+^-ATPase activity [105]. Decreased H^+^-ATPase activity is known to occur upon octanoic acid stress [112] and thus could also occur upon lactic acid stress. The regulation of H^+^-ATPase pumps is supposed to be affected negatively by the Hsp30 chaperone which is induced upon weak-acid stress [113]. It can be concluded that yeast has developed a mechanism that counters energy depletion to prevent futile cell cycling and maintain energy reserves for growth resumption under more favorable conditions [42]. Taking all of this in consideration it is not surprising that upon lactic acid stress many genes involved in ATP-regulation are differently expressed [48].

Metabolic engineering with the aim of obtaining a strain with a larger ATP pool has already been shown to have a positive outcome on lactic acid production in *S. cerevisiae.* The deletion of the gene encoding Gsf2, which is involved in the sorting of glucose transporters to the plasma membrane, resulted in the derepression of glucose-repressed genes involved in the respiratory pathway. In this deletion strain, ATP and NAD^+^ could be generated more efficiently, rescuing the growth defects of the lactic acid producing strain and thereby increasing its productivity [10]. Another example showing the importance of energy-metabolism in lactic acid production with *S. cerevisiae* was provided by Ji-Yoon Song *et al.*, (2016). They replaced the native acetyl-CoA pathway with an ATP-independent (heterologous) alternative. The strain with the introduced ATP-independent pathway showed a 20% increase in lactic acid production [114].

## ROS ACCUMULATION UPON LACTIC ACID STRESS

Production of high titers of lactic acid under anaerobic conditions by metabolically engineered *S. cerevisiae* strains is cumbersome because of ATP depletion [85]. Therefore, production is preferably performed under aerobic conditions. However, there are some inherent disadvantages with aerobic fermentation including higher energy costs and a higher risk of contamination but also increased accumulation of ROS [58]. The formation of ROS is known to cause lipid, protein and nucleic acid oxidative damage. In addition, ROS species have been known to act as second messengers in the induction of a variety of cellular processes [30]. The increase in ROS upon lactic acid stress in aerobic cells is attributed to an increase in superoxide free radical production in combination with the disturbance of the mitochondrial membrane, which results in released iron ions forming lactate-iron complexes [38]. These iron-lactate complexes are known to increase hydroxyl-radical levels via the Fenton reaction [109, 110]. Another possible cause of the increased oxidative stress is the decreased pHi upon lactic acid stress. A drop in pHi favors protonation of superoxide radicals, generating uncharged radical species that are able to diffuse through the plasma membrane, making them much more detrimental [38].

As mentioned previously, the cell reacts to increased ROS formation with an increase GSH formation which is a ROS scavenger [105]. In addition, the levels of the metabolite γ-aminobutyric acid (GABA) also increase drastically [105]. This has been attributed to the role of the GABA shunt from glutamate to succinate, which is responsible for activation of a pathway involved in establishing oxidative stress tolerance [105, 115]. Moreover, activation of the GABA shunt results in increased NADPH levels which are required for GSH regeneration [105, 115].

To counter ROS formation in lactic acid producing *S. cerevisiae* cells under aerobic conditions, metabolic engineering for increased antioxidant levels could be a solution. This has been tried in the past with the development of a strain producing non-natural ascorbic acid [116]. In addition, overexpression of the gene *CTT1*, encoding for a cytosolic catalase has also been shown to increase lactic acid tolerance by alleviating ROS-induced stress [109].

## CELL-ENVELOPE REARRANGEMENTS INDUCED BY LACTIC ACID STRESS

The active expulsion of lactic acid from the cell requires much energy and is also a futile effort if the undissociated acid re-enters at a similar rate at low external pH. Therefore, the cell has developed mechanisms to restrict the amount of passive diffusion and consequently the re-entrance of undissociated lactic acid [31]. One of the proposed mechanisms countering re-entrance of undissociated weak acids is the reinforcement of the cell wall structure to decrease porosity [31, 117]. The decrease of cell-wall porosity upon lipophilic weak acid-stress is a process that is dependent on the cell wall protein Spi1 [118]. However, remodeling of the cell wall by Spi1 does not seem to be equally effective in dealing with small hydrophilic weak acids such as acetic and lactic acid [118]. In the case of hydrophilic weak acid stress, a different response occurs although the desired result (decreased re-entering undissociated acid) appears to be the same. Genes involved in cell-wall regulation that play a role in lactic acid stress tolerance encompass *SED1, DSE2, CTS1, EGT2, SCW11, SUN4* and *YNL300W*. This was shown with functional analysis experiments [48]. Sed1 is a paralog of Spi1 and might play a similar role in the lactic acid stress response as Spi1 plays in the lipophilic weak-acid stress response.

In line with this, the lipid composition of the plasma membrane is also altered upon weak acid stress. The ratio of saturated over unsaturated fatty acids is increased. This happens in combination with a rearrangement of the lipid hydrocarbon tails, and an increase in ergosterol levels that together lead to increased membrane rigidity which results in a decreased amount of undissociated acid that is able to pass the membrane [35, 119, 120]. In addition, this re-organization seems to lead to decreased lipid peroxidation due to a reduction in oxygen-derived free radical attacks [35, 119]. Moreover, the reduced amount of glycans that are found in the cell wall upon lactic acid stress might be a result of altered glucan, mannan and chitin synthesis due to changes in the plasma membrane [35, 121]. Other functions of the plasma membrane that might be affected due to plasma membrane remodeling are stress sensing [122], lipid signaling [123] and protein aggregation [35]. Because the plasma membrane plays a major role in weak acid tolerance, it was not surprising that accumulation of trehalose, which is involved in the protection of proteins and lipids also in membrane structures, increases weak acid tolerance [124, 125].

The targeting of genes involved in cell-envelope rearrangements has also been shown to be relevant for increasing lactic acid titers in a lactic acid producing strain. The deletion of *SAM2,* a gene responsible for the synthesis of the cofactor S-adenosylmethionine (SAM) involved in phospholipid biosynthesis, resulted in higher lactic acid resistance and a higher production of lactic acid in a lactic acid producing *S. cerevisiae* strain.

## LACTIC ACID STRESS IN THE PRESENCE OF OTHER STRESS FACTORS

Obviously, during lactic acid fermentation with *S. cerevisiae*, the negative effect that lactic acid exerts on the cell is not the only inhibiting stress factor in an industrial setting. The fermenter can be contaminated with microorganisms leading to various byproducts that also exert stress on the cells. A second source of stress factors is the composition of the substrate and the inhibitors generated in the pretreatment process. The utilization of pure sugar feedstock does not generate a large excess of inhibitor molecules as in the utilization of cellulosic biomass, but pure sugar streams are relatively expensive. Currently, there is a rising interest in the use of cheap renewable “second-generation” biomass resources as carbon source. These are usually very heterogenous materials either containing a high level of inhibitors in the substrate itself or giving rise to high levels of inhibitors in the pretreatment process [126]. A third potential stress factor in industrial lactic acid fermentations is high temperature. In industrial fermentations, it is preferred to ferment at relatively high temperatures due to the increase in enzyme efficiency and the faster microbial fermentation [127]. However, such high temperatures result in negative effects on the cell [127]. Moreover, because high titers of end product are required in industrial fermentation, very high gravity fermentations are used, resulting in high osmotic pressure [127].

These different types of stress do not only pose individual problems for the cell to deal with. It has been shown that these stress factors often reinforce each other resulting in a very strong combined negative effect on the cell. A good example of this phenomenon is the synergistic effect of acetic acid and lactic acid. The minimum inhibitory concentration (MIC) of acetic acid is 0.6% w/v while that of lactic acid is 2.5% w/v, although there is quite some strain-dependent variation in this respect. However, when 0.05-0.1% acetic acid in combination with 0.2-0.8% w/v lactic acid was added to the medium, a much stronger growth inhibition and reduction in glucose consumption and ethanol production occurred [128]. Another interesting synergy was observed with the effect of ethanol and other alkanols on transport of weak acids. It was shown that when cells were grown in glucose media (i.e. with acetic acid transporters glucose-repressed), the passive influx of undissociated acetic acid increased exponentially upon exposure to ethanol and butanol. The degree of exponential enhancement seemed to correlate with the lipid partition coefficient of the alkanols [129]. In contrast, in medium supplemented with acetic acid, lactic acid or ethanol instead of glucose (i.e. acetic acid transporters not glucose-repressed), acetic acid transport was inhibited in a non-competitive way upon addition of ethanol or other alkanols. The underlying cause was attributed to either conformational changes of the acetic acid transporters or changes in their lipid environment in the plasma membrane caused by the fluidizing activities of the alkanols [129].

Interestingly, the presence of different consecutive stresses on the cell can give rise to a phenomenon called cross-resistance. When the cells are exposed to a particular stress, an adaptive response is triggered resulting in a transient resistance to this specific stress. However, because the general response between different stresses largely coincides, the adapted cells generally are not only resistant to the specific stress applied, but also to other types of stress. For instance, it has been shown that pre-induction of cells in glucose media with mild acid stress using hydrochloric acid (pH=3.5) resulted in more thermotolerant cells [130]. However, when more lipophilic acid was added (50 mM acetic acid at pH=3.5 or excess hydrochloric acid at pH=2.5), an increase in thermotolerance was not observed [130]. Interestingly, it was shown that upon high acid stress, the H^+^-ATPase was more expressed, indicating that a resistance mechanism was indeed induced. However, the resistance mechanism seemed not to be sufficient to counter the negative effects exerted by high acid stress, preventing development of the thermotolerant phenotype [130]. The manner in which the resistance mechanism was activated was proposed to be dependent on a drop in pHi [130]. Another interesting finding was that cells pre-incubated under glucose-starvation conditions were found to be more thermotolerant to high levels of heat stress. This indicates that glucose starvation by itself results in acquisition of thermotolerance [130]. One of the underlying causes might be the drastic decrease in the level of plasma H^+^-ATPase pumps in glucose-starvation media, resulting in a drop of the intracellular pH [50].

It can be concluded that in industrial lactic acid fermentations with *S. cerevisiae* many different individual stresses can have an influence on the fermentation performance. Moreover, these individual stresses can affect each other leading to synergistically detrimental effects. On the other hand, the presence of a particular stressful condition during pre-incubation can lead to resistance against other potential stresses in a later production phase.

## THE DIFFERENCE IN THE STRATEGIES USED TO ACQUIRE LACTIC ACID TOLERANCE IN *ZYGOSACCHAROMYCES BAILII* AND *S. CEREVISIAE*

It is known that some fungal species such as *Z. bailii* [131], *C. krusei* (also named *C. glycerinogenes, P. kudriavzevii* and *I. orientalis*) [56, 132], *Monascus ruber* [132]*, Candida albicans* [133], and *Candida glabrata* [133] are even more lactic acid tolerant than *S. cerevisiae*. The mechanisms underlying lactic acid tolerance vary to a certain extent in different microorganisms. Knowledge about how lactic acid tolerance is established in species that are very lactic acid tolerant can help in finding ways to develop more lactic acid tolerant *S. cerevisiae* strains.

One of the species that has been described extensively for its high acid resistance is *Z. bailii*. One reason for its resistance can be attributed to its ability to use weak acids as carbon source even when glucose is present in the medium [134–136]. In the case of acetic acid tolerance this was attributed due to the presence of an acetate transporter and acetyl-CoA synthetase that are not glucose repressed or inhibited [135, 137, 138]. On the other hand, according to Stratford *et al.* degradation of the weak acid was unlikely to be the main cause for weak acid resistance in *Z. bailii* because of the high diversity in acid structures, the lack of growth restoration in sub-populations, and the cross-resistance between dissimilar acids [139]. Previous work came to the same conclusion [140].

Because weak acids can differ considerably in structure, the resistance mechanisms are likely also diverse. However, some responses to different weak acid stresses in *Z. bailii* appeared to be similar. Upon weak acid stress resistant sub-populations arose that showed cross-resistance. The pHi in these sub-populations was decreased compared to the control. Previously, it was proposed that a drop in the pHi could be the trigger for the induction of resistance mechanisms [130]. However, the drop in pHi was also suggested to be a resistance mechanism by itself. Lower pHi leads to a lower uptake of all weak acids due to an increase in the percentage of undissociated over dissociated acid in the cytosol. The increase in the percentage of undissociated acid in the cell decreases the rate of passive diffusion of the undissociated acid from the outside to the inside of the cell [139]. Since the acidification of the cytosol during weak acid stress did not always lead to more resistant cells, it was suggested that the pHi in these resistant cells was inherently low [139].

Not only a lower inherent pHi, but also drastic lipid rearrangements in the cell membrane and cell wall remodeling have been shown to be major contributors to weak acid tolerance in *Z. bailii* [131, 141]. As in *S. cerevisiae*, the rearrangements of plasma membrane and cell wall are aimed at decreasing the organic acid permeability. However, in *Z. bailii*, the rearrangements are much more extensive [141]. Upon lactic acid stress in *Z. bailii*, phosphocholine levels are reduced significantly in exponential and stationary cells and ergosterol levels are increased, which leads to a considerable increase in membrane rigidity [131]. Additionally, as in *S. cerevisiae*, a decrease in glucans and mannans was observed in the cell wall upon lactic acid stress. Again, this decrease was significantly stronger in *Z. bailii* in contrast to *S. cerevisiae,* suggesting more extensive cell wall rearrangements [131].

In line with this, it was found that *Z. bailli* is able to maintain a more stable pHi upon acetic acid stress than *S. cerevisiae*. The same was shown for *C. krusei* upon lactic acid stress [56]. The more stable pHi in the weak acid resistant yeasts could be explained by the fact that less acid is able to enter the cell because of the inherently lower pHi and/or because of the more rigid cell membrane and cell wall (see above). Another contributing factor could be a higher expression of H^+^-ATPases. Because in *Z. bailli* weak acid tolerance is mainly attributed to its ability to decrease the amount of weak acid that can enter the cell, it may have no apparent need for a weak acid exporter such as Pdr12 [37]. By not utilizing active transport for anion extrusion, the cell can save energy for other metabolic processes. In conclusion, we can infer that *Z. bailli* has developed more efficient weak acid tolerance mechanisms than *S. cerevisiae* and that this is mainly based on keeping the acid better out of the cell. It could be useful to implement similar mechanisms in homolactic *S. cerevisiae* strains to solve the problems created by passive lactic acid uptake and ATP depletion to support its export back to the medium in anaerobic lactic acid fermentation.

## IMPROVING LACTIC ACID TOLERANCE IN *S. CEREVISIAE*

As we have discussed in the previous paragraphs, lactic acid tolerance is a very complex trait, and many different targets therefore seem to be available for possible modification in order to improve this trait. To identify relevant or even additional targets, several approaches have been used, such as a RNAi-mediated genome-wide expression knockdown [13], the utilization of a multi-copy yeast genomic library [29], functional analysis using a deletion strain collection for all nonessential genes [48], monitoring expression changes upon lactic acid stress using DNA microarrays [48] and also adaptive evolution followed by whole-genome sequencing [10, 142]. Another interesting approach was UV-mutagenesis of a lactic acid producing strain followed by selection for maintenance of high pHi via cell sorting [7]. The selection for cells with a higher pHi resulted in identification of mutant strains better adapted to lactic acid stress, and therefore showing better performance in lactic acid production. However, in this case the underlying causative mutations were not identified because the strains were not genome sequenced. Alternative methods that have not been reported yet for analysis and improvement of lactic acid tolerance but could prove to be useful are QTL-mapping by pooled-segregant whole-genome sequence analysis [143, 144] and the utilization of genomic mutagenesis strategies such as EMS mutagenesis [145] or genome shuffling strategies such as whole genome transformation [127, 146], followed by whole-genome sequence analysis to identify the causative genetic modifications.

Improvement of lactic acid tolerance in lactic acid producing *S. cerevisiae* strains has already shown to result in better performance. This has been achieved by both targeted [13, 26, 29] and non-targeted [10, 28] metabolic engineering strategies. In **Table 1**, the reported lactic acid tolerance-related single genetic modifications that have been shown to increase lactic acid production in *S. cerevisiae* are summarized.

**TABLE 1.** List of genetic modifications linked to lactic acid tolerance that have been shown to increase lactic acid production in metabolically engineered *S. cerevisiae* strains.

**Genetic modification**	**Description (SGD)**	**Most likely effect of genetic modification on the cell upon lactic acid stress**	**Lactic acid**				**Genetic modifications in parent strain**	**Ref.**
			**Yield**		**Titer**			
			**Parent strain**	**Modified strain**	**Parent strain**	**Modified strain**		
*GSF2* deletion	ER-located integral membrane protein that promotes secretion of hexose transporters	Increase in ATP and NAD^+^ levels	**Non-buffered media**				*dld1*Δ *jen1*Δ *adh1*Δ *gpd1*Δ *gpd2*Δ *pdc1*Δ*::ldh*	[10]
			58%	72%	16.9g/L	33.2g/L		
*HAA1* overexpression	Transcriptional activator involved in adaptation to weak acid stress; activates transcription of TPO2, YRO2, and other genes encoding membrane stress proteins	Transcriptional activation of lactic acid tolerance related genes	**Non-buffered media**				*dld1*Δ *jen1*Δ *adh1*Δ *gpd1*Δ *gpd2*Δ *pdc1*Δ*::ldh*	[28]
			69%	79%		49 g/L		
			**Buffered medium**					
				80%		112 g/L		
*SSB1* deletion	Ribosome-associated ATPase of the Hsp70 family that has a chaperone-like function	Reparation of DNA damage	**Non-buffered medium**				*pdc1*Δ*::ldh cyb2*Δ*::ldh gpd1*Δ*::ldh*	[13]
			76%	77%	20g/L	26g/L		
			***Buffered medium***					
			72%	73 %	32 g/L	51 g/L		
*SAM2* deletion	SAM synthetase: SAM is a central coenzym playing a role in many intracellular functions such as phospholipid biosynthesis and ROS scavenging	Alternate membrane remodeling and ROS scavenging among other things	**Non-buffered medium**				*pdc1*Δ *pdc5*Δ *pdc6*Δ (LDH expression on multicopy plasmid)	[26]
			87%	88%	65.6 ± 0.9 g/L	69.2 ± 0.6 g/L		
*ESBP6* overexpression	Similar to monocarboxylate permeases. Interacts with a variety of chaperones including Hsp90 which is involved in cell wall porosity	Increase in intracellular pH which is probably due to increased cell wall rigidity	**Non-buffered medium**				*pdc1*Δ*::ldh*	[29]
					4.6 g/L	5.5 g/L		
*SUR1* overexpression	Mannosylinositol phosphorylceramide (MIPC) synthase catalytic subunit; forms a complex with regulatory subunit Csg2; function in sphingolipid biosynthesis is overlapping with that of Csh1	Increase in cell membrane rigidity upon lactic acid stress	**Non-buffered medium**				*adh1*Δ *adh2*Δ *adh3*Δ *adh4*Δ *adh5*Δ *gpd1*Δ *gpd2*Δ *dld1*Δ*::ldh*	[142]
					± 25 g/L	± 30 g/L		
*ERF2* deletion	Subunit of palmitoyltransferase which is required for posttranslational modification of several proteins such as Ras1, 2, Rho2 and 3 and Gpa1 and 2	Membrane localization of proteins involved in lactic acid tolerance is altered	**Non-buffered medium**				*adh1*Δ *adh2*Δ *adh3*Δ *adh4*Δ *adh5*Δ *gpd1*Δ *gpd2*Δ *dld1*Δ*::ldh*	[142]
					± 25 g/L	± 30 g/L		

## INTELLECTUAL PROPERTY ON MICROBIAL LACTIC ACID FERMENTATION WITH YEAST

The importance of improving lactic acid tolerance for microbial lactic acid production has been recognized for many years. Hence, multiple patent applications have been submitted on genetic modifications of genes affecting lactic acid tolerance and on the resulting lactic acid tolerant strains. In **Table 2**, an overview has been provided.

**TABLE 2.** List of patent applications on genetic modifications affecting lactic acid tolerance and the resulting lactic acid tolerant strains.

**Patent summary**	**Description**	**Owners**	**Priority Date**	**Issued in**	**Ref.**
A method of producing lactic acid with Pdc-, C_2_-independent acid tolerant strains expressing a heterologous *LDH* gene in aerobic conditions	Acid tolerant strain was generated using adaptive evolution with growth at low pH as a selection criterion.	Tate & Lyle Ingredients Americas LLC	November 20, 2003	World (WO 2005/052174) USA (US 71799303)	[147]
A method for increasing tolerance to organic acids and low pH in yeast by overexpression of a H^+^-ATPase *(PMA1)*	Pma1 (SGD): Plasma membrane P2-type H^+^-ATPase; pumps protons out of cell; major regulator of cytoplasmic pH and plasma membrane potential	Tate & Lyle Ingredients Americas LLC	June 6, 2007	World (WO 2008/153890) USA (US 93333807)	[58]
Yeast in which the *SED1* gene is exchanged with an *LDH* expression cassette	Sed1 (SGD): Major stress-induced structural GPI-cell wall glycoprotein; associates with translating ribosomes, possible role in mitochondrial genome maintenance. Sed1 has previously been associated with lactic acid tolerance [27].	Toray Industries Inc.	December 7, 2007	USA (US 8859260) Japan (JP 2008072129)	[148]
Lactic acid tolerant strains (NITE BP-1087 NITE BP-1088 NITE BP-1089 NITE BP-1189 NITE-BP 1190)	EMS mutagenized SU042 parent strain (*Limulus polyphemus D-LDH* introduced) and HI003 parent strain (*Xenopus laevis L-LDH* introduced) selected for lactic acid tolerant phenotype	Toray Industries Inc.	April 28, 2011	World (WO 2012/147903) Japan (JP 2012016544) USA (US 9217165)	[149]
Overexpression of the lactic acid transporter (Ady2) in a lactic acid producing strain with inhibition of Cyb2 and Pdc activity	See chapter “The role of acetate permease Ady2 in lactate transport“	Samsung Electronics Co. Ltd.	Februari 5, 2013	USA (US 9353388) South Korea (KR 20130012938)	[150]
Lactic acid tolerant strain (12532BP strain (KCTC))	EMS mutagenized CEN.PK2-1D strain (*pdc1Δ::ldh cyb2Δ::ldh gpd1::ldh*) selected for a lactic acid tolerant phenotype	Samsung Electronics Co. Ltd.	Februari 13, 2014	South Korea (KR 20140016790) USA (US 9617570)	[151]
Lactic acid resistant yeast comprising genetic mutation that reduces Fps1 activity	Fps1 (SGD): Aquaglyceroporin, plasma membrane channel; involved in efflux of glycerol and in transport of acetate; key factor in maintaining redox balance by mediating passive diffusion of glycerol; phosphorylated by Hog1 and Slt2 MAPK; regulated by Rgc1 and Ask10, which are regulated by Hog1 phosphorylation under osmotic stress; phosphorylation by Ypk1 required to maintain an open state.	Samsung Electronics Co. Ltd.	June 23, 2014	USA (US 2015/0368306) South Korea (KR 20140076636)	[152]
A recombinant acid-tolerant yeast strain comprising increased activity of the Rck1/2 enzyme	Rck1/2 (SGD): Protein kinase involved in oxidative stress response	Samsung Electronics Co. Ltd.	July 24, 2014	USA (US 2016/0024484) South Korea (KR 20140094159)	[153]
Lactic acid tolerant strain, wherein the yeast strain has enhanced Msn2 activity	Msn2 (SGD): Stress-responsive transcriptional activator; activated in stochastic pulses of nuclear localization in response to various stress conditions; binds DNA at stress response elements of responsive genes	Samsung Electronics Co. Ltd.	July 28, 2014	USA (US 2016/0024537) South Korea (KR 20140096013)	[154]
Lactic acid resistant yeast showing improved activity of the *SUL1* gene product	Sul1 (SGD): High affinity sulfate permease of the SulP anion transporter family; sulfate uptake is mediated by specific sulfate transporters Sul1 and Sul2, which control the concentration of endogenous activated sulfate intermediates	Samsung Electronics Co. Ltd.	May 12, 2015	South Korea (KR 20150066250) USA (US 2016/0333380)	[155]
Lactic acid resistant yeast showing improved activity of the *STR3* gene product	Str3 (SGD): Peroxisomal cystathionine beta-lyase; converts cystathionine into homocysteine; may be redox regulated by Gto1	Samsung Electronics Co. Ltd.	May 12, 2015	South Korea (KR 20150066250) USA (US2016/0333380)	[155]
Lactic acid resistant yeast showing improved activity of the *HXT7* gene product	Hxt7 (SGD): High-affinity glucose transporter; member of the major facilitator superfamily, nearly identical to Hxt6, expressed at high basal levels relative to other HXTs, expression repressed by high glucose levels; HXT7 has a paralog, HXT4, that arose from the whole genome duplication	Samsung Electronics Co. Ltd.	May 12, 2015	South Korea (KR 20150066250) USA (US2016/0333380)	[155]
Lactic acid resistant yeast showing improved activity of the *ERR1* gene product	Err1 (SGD): Putative phosphopyruvate hydratase	Samsung Electronics Co. Ltd.	May 12, 2015	South Korea (KR 20150066250) USA (US2016/0333380)	[155]
Lactic acid resistant yeast showing improved activity of the *GRX8* gene product	Grx8 (SGD): Glutaredoxin that employs a dithiol mechanism of catalysis; monomeric; activity is low and null mutation does not affect sensitivity to oxidative stress; GFP-fusion protein localizes to the cytoplasm; expression strongly induced by arsenic	Samsung Electronics Co. Ltd.	May 12, 2015	South Korea (KR 20150066250) USA (US2016/0333380)	[155]
Lactic acid resistant yeast showing improved activity of the *MXR1* gene product	Mxr1 (SGD): Methionine-S-sulfoxide reductase; involved in the response to oxidative stress; protects iron-sulfur clusters from oxidative inactivation along with MXR2; involved in the regulation of lifespan	Samsung Electronics Co. Ltd.	May 12, 2015	South Korea (KR 20150066250) USA (US2016/0333380)	[155]
Lactic acid resistant yeast showing improved activity of the *GRE1* gene product	Gre1 (SGD): Hydrophilin essential in desiccation-rehydration process; stress induced (osmotic, ionic, oxidative, heat shock and heavy metals); regulated by the HOG pathway; GRE1 has a paralog, SIP18, that arose from the whole genome duplication	Samsung Electronics Co. Ltd.	May 12, 2015	South Korea (KR 20150066250) USA (US2016/0333380)	[155]
Lactic acid resistant yeast showing improved activity of the *MRK1* gene product	Mrk1 (SGD): Glycogen synthase kinase 3 (GSK-3) homolog; one of four GSK-3 homologs in *S. cerevisiae* that function to activate Msn2-dependent transcription of stress responsive genes and in protein degradation; MRK1 has a paralog, RIM11, that arose from the whole genome duplication	Samsung Electronics Co. Ltd.	May 12, 2015	South Korea (KR 20150066250) USA (US2016/0333380)	[155]
Lactic acid resistant yeast showing improved activity of the *AAD10* gene product	Aad10 (SGD): Putative aryl-alcohol dehydrogenase	Samsung Electronics Co. Ltd.	May 12, 2015	South Korea (KR 20150066250) USA (US2016/0333380)	[155]
An acid-tolerant, lactic acid producing yeast strain that has a genetic modification increasing Aur1 activity	Aur1 (SGD): Phosphatidylinositol:ceramide phosphoinositol transferase; required for sphingolipid synthesis; can mutate to confer aureobasidin A resistance; also known as IPC synthase	Samsung Electronics Co. Ltd.	July 28, 2015	USA (US 10053714) South Korea (KR 20150106771)	[156]
An acid-tolerant, lactic acid producing yeast strain that has a genetic modification that increases activity of an enzyme that introduces a double bond to a fatty acyl site of a fatty acyl-CoA (Ole1)	Ole1 (SGD): Delta(9) fatty acid desaturase; required for monounsaturated fatty acid synthesis and for normal distribution of mitochondria	Samsung Electronics Co. Ltd.	July 28, 2015	USA (US 10053714) South Korea (KR 20150106771)	[156]
An acid-tolerant, lactic acid producing yeast strain that has a genetic modification that decreases activity of an enzyme that catalyzes formation of triacylglycerol from diacylglycerol (Dga1/Lro1)	Dga1/Lro1 (SGD): Diacylglycerol acyltransferases; catalyzes the terminal step of triacylglycerol (TAG) formation, acylates diacylglycerol using acyl-CoA as an acyl donor; Lro1 and Dga1 can O-acylate ceramides; localized to lipid particles	Samsung Electronics Co. Ltd.	July 28, 2015	USA (US 10053714) South Korea (KR 20150106771)	[156]

Not only genetic modifications conferring better lactic acid tolerance and covering lactic acid tolerant strains have been patented in regard to lactic acid fermentation with yeast. Other items that have been patented comprise process methodologies [157–162], second-generation lactic acid producing yeast strains [163], heterologous *LDH* sequences [164–167] and methods to improve the performance of a yeast population for lactic acid production [168]. In addition, other genetic modifications not related to enhancing lactic acid tolerance but rather to improving the performance of lactic acid producing strains in other ways have been patented. These modifications comprise genetic mutations that alter the glycolytic flux (due to decreased Rgt1 activity [169], increased Gcr1/2 activity [170], deletion/disruption of *STD1/MTH1* [170], or overexpression of sugar transporters [171]) and alterations in NADH and NADPH metabolism (due to *NDE1/2* deletion [172] or enhancement of Zwf1 activity [173]). Moreover, patent applications have been filed on items such as the introduction of a heterologous ATP-citrate lyase, genetic modifications enhancing the pyruvate biosynthetic pathway [174] and modifications improving pyruvate availability [175]. Other examples of genetic modifications that have been patented in lactic acid producing strains are the deletion of *CYB2* to decrease lactic acid utilization under aerobic conditions [176], constitutive expression of *HAP4* [177], a genetic modification conferring enhanced Edc1/2 activity [178] and a genetic modification reducing Rim15 and Igo2 activity [179].

## CONCLUSIONS

We can conclude that lactic acid tolerance is an important trait to consider in developing a lactic acid producing microbial cell factory. Although lactic acid tolerance appears to be a very complex trait, there is already substantial knowledge on the underlying mechanisms and the responses of the cell to lactic acid stress, not only in *S. cerevisiae* but also in other microorganisms. In *S. cerevisiae,* genes involved in lactic acid tolerance have been linked to various processes such as transcriptomic regulation, pHi homeostasis, anion transport, ROS scavenging, cell-envelope rearrangements and amino acid, iron and energy metabolism. However, just a handful of causative genes have been evaluated in lactic acid producing strains for improvement of performance. In the future, more causative genes should be tested, especially in industrial strains already producing a high lactic acid titer. Moreover, since it has been shown that a combination of multiple genetic modifications can be even more beneficial, this approach should be applied more systematically to develop strains with superior performance for lactic acid production [27].

## AUTHOR CONTRIBUTIONS

AP: Formal analysis, writing - original draft; MRF and JMT: Formal analysis, writing - original draft.

## Abbreviations:

GABA: – γ-aminobutyric acid,
GSH: – glutathione,
pHi: – intracellular pH,
PLA: – poly-lactic acid,
ROS: – reactive oxygen species,
SNP: – single nucleotide polymorphism.
